# Differential Effects of Corneal Biomechanics on Superficial and Deep Vessel Density and Their Association with Central Visual Function in Glaucoma Patients with Myopia

**DOI:** 10.3390/jcm14186515

**Published:** 2025-09-16

**Authors:** Kyoung Ohn, Younhea Jung, Hae-Young Lopilly Park

**Affiliations:** 1 Department of Ophthalmology, Yeouido St. Mary’s Eye Hospital, College of Medicine, The Catholic University of Korea, Seoul 07345, Republic of Korea; kyoungohn@gmail.com (K.O.); write2une@catholic.ac.kr (Y.J.); 2 Department of Ophthalmology, Seoul St. Mary’s Hospital, College of Medicine, The Catholic University of Korea, 222, Banpodaero, Seochogu, Seoul 06591, Republic of Korea

**Keywords:** corneal biomechanical properties, glaucoma, Corvis ST, optical coherence tomography angiography (OCTA), central visual field, myopia

## Abstract

**Background/Objectives:** The purpose of this study was to investigate corneal biomechanical properties assessed with Corvis ST, structural features of myopia, and vessel density (VD) measured by optical coherence tomography angiography (OCT-A) and their associations with central visual function in myopic glaucoma patients. **Methods:** Forty-two eyes of 42 glaucoma patients with myopia without retinal lesions were subjected to analysis. Corvis ST was performed to measure the biomechanical properties of the eyeball. Superficial (retinal) and deep (choroidal) VDs in the peripapillary and macular regions were assessed using OCT-A, and retinal nerve fiber layer (RNFL) thickness was measured with OCT. The disc–foveal angle, disc torsion, and β-zone peripapillary atrophy (PPA) area were obtained from disc and retinal photography. Swedish interactive thresholding algorithm (SITA) 24-2 visual field (VF) testing was used to evaluate the function within the central 12 points. **Results:** A worse mean deviation (MD) from SITA 24-2 and higher whole-eye movement maximum from Corvis ST, representing deformable corneas, were associated with lower superficial peripapillary VD. A lower A1 deflection amplitude from Corvis ST, representing stiffer corneas, was associated with lower deep peripapillary VD. The sensitivity of the central 12 VF points was significantly associated with a larger disc–foveal angle, lower superficial peripapillary VD, and lower HC deformation amplitude from Corvis ST. **Conclusions:** Our preliminary findings suggest that more compliant corneas were associated with lower superficial VD, whereas stiffer corneas were associated with lower deep VD and central scotoma.

## 1. Introduction

Corneal biomechanical properties in glaucoma have received increasing attention because they may reflect structural vulnerabilities of the posterior sclera and optic nerve head (ONH), which play a role in the development and progression of glaucoma [1,2,3,4,5,6]. The biomechanical characteristics of ocular tissues influence their response to elevated intraocular pressure (IOP) and may explain individual differences in susceptibility to glaucomatous damage.

Previous studies reported that glaucoma patients showed significantly less deformable corneas than controls, even after adjusting for IOP, central corneal thickness (CCT), age, and axial length [7,8,9], suggesting stiffer corneas in glaucomatous eyes. Notably, within glaucoma populations, relatively more deformable corneas showed a faster rate of visual field progression in patients with primary open-angle glaucoma (POAG) and normal-tension glaucoma (NTG), indicating that biomechanical weakness may increase susceptibility to disease progression [10,11]. Moreover, the corneal biomechanics are different in various types of glaucoma. The corneas of NTG patients are softer than those of high-tension glaucoma patients and controls [12,13,14,15], while eyes with pseudoexfoliative glaucoma show stiffer corneas with reduced deformability compared to those with POAG and controls [16,17].

Furthermore, the relation between corneal biomechanics and ONH structure has been described [18,19]. Patients with lower corneal deformation amplitude (DA) showed greater depth of the lamina cribrosa (LC), greater cup area, deeper cups, and smaller peripapillary atrophy (PPA) than those with higher corneal DA [20]. A greater posterior LC curvature was linked to lower corneal stiffness parameters in younger patients with NTG [21]. DA was correlated with the PPA area and disc tilt ratio in glaucoma [22]. However, the relationship between corneal biomechanics and VD has not been described.

Axial elongation in myopic eyes leads to various structural changes. The stretching and distortion of the posterior sclera and the ONH result in damage to the optic nerve fibers, making myopic eyes more susceptible to glaucomatous damage [23,24,25,26,27,28]. Consequently, myopia, particularly high myopia, is known as an important risk factor for glaucoma development.

Notably, central VF damage is frequently observed even in the early stages of glaucomatous myopic eyes and significantly impacts patients’ quality of life [29,30,31,32]. Both structural and vascular changes have been reported to be linked to central visual function. Hemodynamic instability and blood flow insufficiency have been widely reported as contributing factors [33,34,35,36,37]. Additionally, changes in the posterior scleral profiles have been known to be related to the development of central scotoma in myopic patients [38,39,40]. However, the factors that contribute to central visual function in myopic glaucoma have not yet been clearly identified.

Previous histological studies have demonstrated that in myopic eyes, the collagen fibers of the sclera elongate and deform due to stretching, which alters the biomechanical characteristics of the whole eyeball [41,42]. Moreover, studies have shown that the cornea, sclera, peripapillary ring, and LC all share similar extracellular matrix (ECM) components in their composition [43]. These findings suggest that the corneal biomechanical properties may, to some extent, represent biomechanical properties of adjacent tissues. Furthermore, axial elongation-related thinning of the retina, choroid, and sclera may affect peripapillary VD around the ONH [44,45].

Based on these considerations, we hypothesized that scleral biomechanical properties affect axial elongation, which in turn influences peripapillary VD and central VF defects in glaucoma. Therefore, corneal biomechanics may serve as a surrogate marker reflecting these processes. In this study, we performed optical coherence tomography angiography (OCT-A) and Corvis ST in myopic glaucoma patients to investigate the association between corneal biomechanics and VD in relation to central scotoma.

## 2. Materials and Methods

Study Participants: This cross-sectional, retrospective study was approved by the Institutional Review Board of Yeouido St. Mary’s Hospital and conducted in accordance with the tenets of the Declaration of Helsinki (IRB No. SC23RISI0094).

This study retrospectively reviewed the clinical data of patients who visited Yeouido St. Mary’s Eye Hospital (Seoul, Republic of Korea) from July 2022 to December 2022. Patients with open-angle glaucoma and myopia were included. Myopia was defined based on an axial length of ≥24 mm measured by ocular biometry (IOLMaster; Carl Zeiss Meditec, Jena, Germany).

All subjects underwent complete ophthalmic examinations including slit-lamp examination, gonioscopy, Goldmann applanation tonometry, ultrasound pachymetry (Tomey, Nagoya, Japan), and dilated stereoscopic examination of the optic disc (Canon, Tokyo, Japan). Peripapillary retinal nerve fiber layer (RNFL) thicknesses were obtained using spectral-domain optical coherence tomography (OCT) (Cirrus HD- OCT; Carl Zeiss Meditec, Jena, Germany).

All participants underwent standard automated perimetry with the Swedish interactive thresholding algorithm (SITA) 24-2 (Humphrey Visual Field Analyzer; Carl Zeiss Meditec). Peripapillary and macular VDs were evaluated with swept-source OCT-A (DRI OCT Triton; Topcon, Tokyo, Japan).

Open-angle glaucoma was diagnosed when both glaucomatous VF abnormalities and corresponding optic disc changes were present. Disc changes included diffuse or focal neuroretinal rim thinning, rim notching, an increased vertical cup-to-disc ratio (≥0.7 or ≥0.2 greater than that for the fellow eye), or RNFL loss. VF abnormalities were required to be reproducible on at least two reliable SITA 24-2 examinations (fixation losses < 20%, false negatives < 15%, false positives < 15%) and were defined as positive when two or more of the following were satisfied: (1) a cluster of ≥3 points with *p* < 0.05 on the pattern deviation map in a single hemifield, including at least 1 point with *p* < 0.01, or a cluster of ≥2 points with *p* < 0.01; (2) glaucoma hemifield test outside normal limits; or (3) PSD exceeding the 95% confidence limits.

Corvis ST: The corneal biomechanical properties were assessed with Corvis ST (Oculus Optikgeräte GmbH, Wetzlar, Germany). After proper alignment, an air puff of 25 kPa was automatically applied from a distance of 11 mm. The cornea subsequently passed through the first applanation (A1), reached the highest concavity (HC), and then recovered through the second applanation (A2) to its baseline shape. The central 8.5 mm corneal deformation was documented by an ultra-high-speed Scheimpflug camera, which acquired 140 frames within 30 ms at a resolution of 640 × 480 pixels. The average value from three consecutive trials was used for statistical analysis.

Among the Corvis ST parameters, deformation amplitude (DA) was defined as the total vertical displacement of the corneal apex at HC, whereas deflection amplitude was defined as the displacement of the cornea corrected for whole-eye movement. Definitions of all Corvis ST parameters analyzed in this study are provided in Table S1.

OCT-A: Swept-source OCT-A imaging was performed with the DRI OCT Triton device (Topcon Inc., Tokyo, Japan), which operates at a 1050 nm wavelength and captures 100,000 A-scans per second, providing an axial resolution of 8 μm. All scans covered a 4.5 × 4.5 mm field and incorporated active eye tracking to reduce motion artifacts. Only images with a quality index ≥ 40 were accepted for further analysis. Blood flow data were processed using the OCTA ratio analysis (OCTARA) algorithm, which automatically segments the optic nerve head and macular regions into four en face layers.

For the peripapillary region, the superficial plexus corresponded to the RPC layer (ILM to RNFL). Superficial VD was subdivided into temporal and nasal sectors using a vertical reference line 90° from the fovea–disc axis. The deep peripapillary slab encompassed signals from the RPE to the outer scleral boundary, primarily representing choroidal vessels. In this study, superficial VD referred to the retinal vascular plexus, whereas deep VD corresponded to the choroidal vasculature.

Deep peripapillary VD was quantified within the β-zone PPA, which was manually outlined along with the disc margin using ImageJ software, version 1.53t (National Institutes of Health, Bethesda, MD, USA; 2022). Eyes without β-zone PPA were excluded. Images were binarized with the ImageJ mean threshold algorithm, assigning vessel pixels as white and background pixels as black. Deep peripapillary VD was expressed as the proportion of vessel pixels within the β-zone PPA relative to the total PPA area.

For the macula, the superficial capillary plexus was defined from 2.5 μm below the ILM to 15.5 μm beneath the IPL/INL junction, and the deep plexus extended from 15.5 μm to 70 μm below the IPL/INL.

VD measurements were derived in ImageJ after 8-bit binarization with the built-in mean thresholding algorithm. Vessel presence was defined as a score ≥ 5, and VD was calculated as the vessel area divided by the total area of interest. Each image was independently reviewed by two masked investigators (K.O. and Y.J.), and low-quality scans (e.g., low signal, segmentation errors, fixation loss, or motion artifacts) were excluded.

Structural parameters of the ONH: The optic disc torsion, disc–foveal angle, and β-zone PPA area were evaluated from color fundus photographs and red-free RNFL images. All images were independently assessed in random order by two authors (K.O. and Y.J.), who were masked to clinical data. These parameters were quantified on the photographs using ImageJ software (version 1.40; National Institutes of Health), following previously published methods [24,26,40,46].

Optic disc torsion was defined as the deviation of the disc’s long axis from the vertical meridian; positive values indicated inferotemporal torsion, whereas negative values indicated superotemporal torsion. The disc–foveal angle was defined as the angle between the disc–fovea reference line and a horizontal line through the disc center, with positive values denoting a fovea located below the disc and negative values denoting a fovea above it [40,46]. The β-zone PPA was defined as an inner crescent of chorioretinal atrophy exposing the sclera and underlying choroidal vessels on fundus photographs. Disc and PPA margins were manually traced with a mouse-driven cursor, and the pixel area of the β-zone PPA was then calculated using ImageJ software (version 1.40; National Institutes of Health).

OCT: Optic disc imaging was performed with the Cirrus HD-OCT system (software v6.0) using the Optic Disc Cube 200 × 200 protocol, which incorporates automated RNFL analysis. Image quality was reviewed by an experienced examiner masked to patient information and other test outcomes. Average and quadrant RNFL thicknesses were derived from the RNFL algorithm, while GCIPL thicknesses (average and six sectors: superotemporal, superior, superonasal, inferotemporal, inferior, and inferonasal) were obtained using the built-in software. Only scans with adequate focus and centration, with signal strength ≥ 7, and free of motion or segmentation artifacts were included in the analysis.

Definition of Central Scotoma: All participants underwent standard automated perimetry (SAP) using the 24-2 SITA Standard program with a Humphrey Field Analyzer II 750i (Carl Zeiss Meditec). Raw 24-2 data were extracted, and each test point was analyzed based on threshold sensitivity values. Global indices including the mean deviation (MD) and pattern standard deviation (PSD) were also evaluated. For analysis, the 12 central points within the central 10° of the 24-2 test were selected [47].

To identify central visual field defects (CVFDs), we reviewed the first consecutive SITA 24-2 results. CVFDs were defined as defects located within the central 10° on the pattern deviation probability map, meeting one of the following: a cluster of ≥3 points with *p* < 0.05 including ≥1 point with *p* < 0.01, or ≥2 contiguous points with *p* < 0.01. Subjects were included if such defects were present in the superior or inferior hemifield of the central 10°, regardless of additional peripheral loss.

Central visual function was further evaluated by calculating retinal sensitivity from the 12 central points of the SITA 24-2 test. Sensitivity values in decibels were converted to a linear scale using the formula [dB = 10 log(1/Lambert)].

The following exclusion criteria were applied to minimize the influence of confounding factors: (1) axial length > 30 mm, (2) retinal diseases such as diabetic retinopathy or myopic macular degeneration, (3) prior ocular trauma or surgery other than uncomplicated cataract extraction, (4) optic neuropathies other than glaucoma, or (5) systemic or neurological disorders potentially affecting VF results.

Statistical analysis: Group comparisons for continuous variables were conducted using Student’s *t*-test or the Mann–Whitney U-test, while categorical variables were analyzed with the chi-square test. Factors related to vessel density and central scotoma were explored through univariate and multivariate regression models. Associations between parameters were further examined using Spearman’s correlation coefficient. All statistical procedures were performed with SPSS software (version 26.0; IBM Corp., Armonk, NY, USA), and a two-sided *p* value < 0.05 was considered statistically significant.

## 3. Results

The baseline characteristics of the study participants were stratified according to their A1 deflection amplitude (Table 1). The participants were classified into high- and low-deflection groups, with a cut-off value of 0.095 (a lower value indicates a stiffer cornea). The high-deflection group tended to be older than the low-deflection group. In addition, the high-deflection group exhibited higher deep peripapillary vessel density than the low-deflection group. Axial length, MD, PSD of the VF24-2, RNFL thickness, and macular VD parameters did not differ significantly between the groups.

Next, we systematically explored the correlation between Corvis ST parameters and various other parameters of the study participants (Table 2). The subjects’ age, central corneal thickness, intraocular pressure, structural parameters of the optic nerve head, chorioretinal vessel densities, and parameters of the VF were subjected to the analysis. Peripapillary VD showed significant correlations with certain Corvis ST parameters. Notably, both higher A1 deformation amplitude and whole-eye movement max showed significant correlations with a decreased superficial temporal VD, while we observed a trend in the opposite direction for the deep peripapillary VD. Higher A1 deflection amplitude exhibited a correlation with higher deep peripapillary VD. For clarity, only significant results are presented in Table 2, Table 3, Table 4, Table 5 and Table 6, while the full set of correlations, including non-significant results, is provided in the Supplementary Materials (Tables S2–S6).

**Figure 1:**
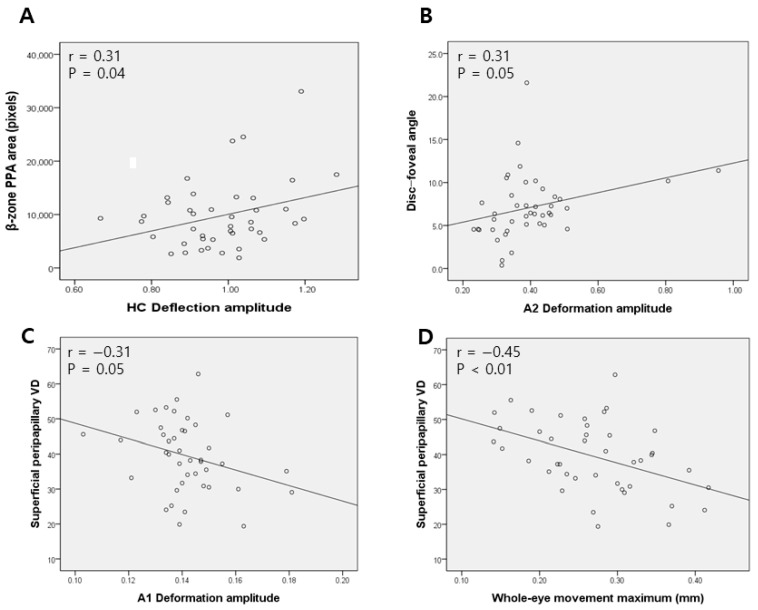
Scatterplots representing correlations between Corvis ST parameters and (**A**) peripapillary atrophy (PPA), (**B**) disc–foveal angle, and (**C**,**D**) superficial peripapillary vessel density (VD).

Figure 1A,B illustrate the quantitative associations between Corvis ST parameters and either the PPA area or superficial peripapillary temporal VD. HC deflection amplitude showed a significant positive correlation with the PPA area. A2 deformation amplitude showed a significant positive correlation with the disc–foveal angle (r = 0.31, *p* = 0.04 and r = 0.31, *p* = 0.05, respectively). Figure 1C,D depict negative correlations between both higher A1 deformation amplitude and whole-eye movement max and the superficial temporal VD (r = −0.31, *p* = 0.05 and r = −0.45, *p* < 0.01, respectively).

We conducted additional investigations to assess the impact of superficial peripapillary temporal VD on various parameters (Table 3). In a multivariate model that incorporated the patients’ age and parameters that exhibited significant associations with superficial temporal VD in univariate analysis, worse mean deviation (MD) of the SITA 24-2 and higher whole-eye movement maximum were associated with lower superficial peripapillary VD (B = −42.340 [95% CI: −76.404 to −8.276], *p* = 0.016).

**Figure 2:**
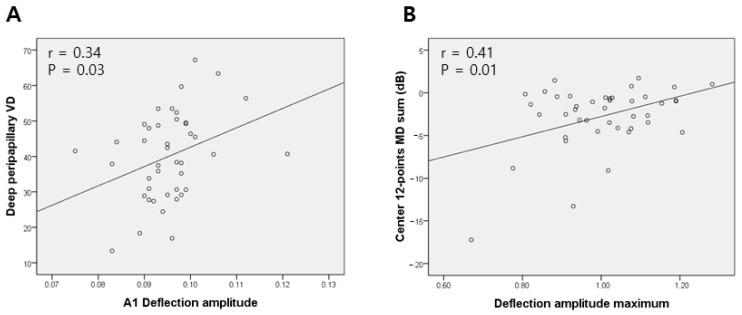
Scatterplots representing correlations between (**A**) A1 deflection amplitude and deep peripapillary vessel density (VD); (**B**) deflection amplitude maximum and central visual field defect.

Figure 2 illustrates the quantitative associations between Corvis ST parameters and either the deep peripapillary VD or the central visual field defect. A1 deflection amplitude showed a positive correlation with the deep peripapillary VD (Figure 2A; r = 0.34, *p* = 0.03). Similarly, deflection amplitude max showed a significant positive correlation with the central 12-point MD sum (Figure 2B; r = 0.41, *p* = 0.01).

The positive correlation between A1 deflection amplitude and deep peripapillary VD persisted in the univariate analysis (Table 4). In a multivariate model that accounted for the patients’ age, intraocular pressure (IOP), and axial length, lower A1 deflection amplitude was associated with lower deep peripapillary VD (B = 745.458 [95% CI: 190.572–1.3 × 10^3^], *p* = 0.010).

Furthermore, we explored the influence of several factors on central VF defects. These relationships were examined using two variables, central scotoma presence and the center 12-point MD from SITA 24–2. Lower superficial peripapillary vessel density (VD) and higher whole-eye movement (WEM) were associated with the presence of central scotoma in the univariate analysis. However, in the multivariate model accounting for the patients’ intraocular pressure (IOP) and axial length, neither superficial peripapillary VD nor WEM was significantly related (Table 5).

A larger disc–foveal angle, lower superficial peripapillary VD, lower HC deformation amplitude, and lower HC deflection amplitude were associated with a lower center 12-point MD from SITA 24-2 in the univariate analysis (Table 6). In multiple models that accounted for different variables, the superficial peripapillary VD and disc–foveal angle continued to have a significant impact on the center 12-point MD from SITA 24-2 (B = 0.123 [95% CI: 0.019–0.226], *p* = 0.022 and B = −0.411 [95% CI: −0.679–−0.143], *p* = 0.004, respectively). A lower HC deflection amplitude and lower HC deformation amplitude were associated with a lower center 12-point MD from SITA 24-2 (B = 15.055 [95% CI: 1.694–28.415], *p* = 0.028 and B = 12.442 [95% CI: −0.303–25.187], *p* = 0.055, respectively).

## 4. Discussion

To the best of our knowledge, this is the first study to investigate the relationships between vessel density and corneal biomechanical properties measured by Corvis ST in glaucoma patients with myopia. Our findings demonstrate distinct associations between superficial and deep peripapillary VD and corneal biomechanics, emphasizing the differing effects on these vascular layers.

Our results showed that greater corneal deformability, as indicated by higher HC deflection amplitude, was significantly associated with a larger β-zone PPA area and a larger disc–foveal angle, both of which are structural changes due to axial elongation in myopia. Furthermore, both higher A1 deformation amplitude and whole-eye movement maximum, indicating compliant corneas, correlated significantly with reduced superficial peripapillary VD. These findings suggest that the biomechanical properties of ocular tissue influence peripapillary vascular dynamics differently depending on the tissue’s deformability. Specifically, in more compliant scleras, even with similar degrees of myopia (axial length), the peripapillary region undergoes greater mechanical stretching, resulting in a larger beta-zone PPA area and a wider disc–foveal angle, both indicative of increased retinal expansion. Consequently, superficial peripapillary VD, corresponding to the radial peripapillary capillary (RPC) layer between the internal limiting membrane (ILM) and the RNFL, is reduced due to the mechanical stretching of the retinal vasculature in this region [37]. These observations are consistent with previous studies reporting that high myopia is associated with reduced VD in the RPC layer of the optic disc [48,49,50]. Taken together, these findings may indicate that myopic eyeball expansion is associated with the structural vulnerability of myopic eyes.

However, more importantly, stiffer corneas, represented by lower A1 deflection amplitudes, were associated with reduced deep peripapillary VD. In addition, stiffer corneas, represented by lower deflection amplitude maxima, were associated with lower central 12-point MD sums, which represent central visual function. Since the deep peripapillary VD represents vessels within the sclera and particularly in the deep choroid extending into the LC, increased scleral stiffness may exert greater compressive force on the peripapillary sclera and deep vessels, impairing microcirculation. These findings suggest that tissue stiffness may play a role in influencing the compression of these specific structures in glaucoma patients with myopia. This indicates that myopic eyes possess not only structural vulnerability due to eyeball expansion but also vascular instability that is associated with the biomechanical properties of the peripapillary sclera, potentially leading to central visual damage. There is growing evidence that structural changes in the posterior pole may affect vascular factors in glaucoma. Shin et al. reported that peripapillary scleral deformation was significantly associated with choroidal microvascular dropout in glaucoma [45]. The peripapillary choroidal microvasculature is supplied by the short posterior ciliary arteries, which penetrate the sclera near the entrance of the optic nerve at the level of the lamina [51]. They hypothesized that scleral deformation at the junction with the lamina cribrosa around the ONH may compress the adjacent short posterior ciliary arteries, reducing blood flow to the ONH and potentially contributing to glaucomatous damage. Scleral deformation results in stiffening of the peripapillary scleral tissue, which may have been observed as a stiff cornea using Corvis ST in the present study, in conjunction with our previous report.

The reduction in deep peripapillary VD in stiffer tissues may have contributed to central visual function damage. Kamalipour et al. reported that lower corneal hysteresis is associated with a higher risk of central VF progression, suggesting that corneal biomechanics may contribute to central vision function damage [52]. As an extension of the previous study, our data showed a significant association between a stiffer cornea and more severe central VF defects, potentially due to ischemia caused by reduced choriocapillaris flow from the compression of a stiffer sclera in the PPA area and papillomacular nerve fiber damage resulting from scleral changes during the progression of myopia (ischemic or structural compromise in the deeper retinal and choroidal layers). Both structural changes and vascular insufficiency may lead to central VF damage in myopic eyes. This aligns with previous studies suggesting that deep peripapillary VD is a critical marker of central scotoma formation in myopic glaucoma eyes. In our previous work, we found that central vision impairment was associated with low deep peripapillary VD in glaucomatous myopic eyes [33,37].

Although there have been no studies reporting the relationship between corneal biomechanics measured by Corvis ST and vascular factors in glaucoma, Mohammadzadeh et al. reported that lower corneal hysteresis was associated with faster rates of decline in ONH capillary density. However, their analysis was limited to the superficial vascular layer (between the internal limiting membrane and the posterior boundary of the RNFL). In addition, Chuangsuwanich et al. further reported that stiffer scleral tissue was linked to lower compressive strain and improved oxygenation, suggesting a protective biomechanical effect [53]. However, their findings were based on mathematical modeling rather than direct biomechanical measurements.

In myopic eyes, axial elongation is closely related to posterior scleral remodeling, a process characterized by significant alterations in the composition and structure of extracellular matrix (ECM) components [54]. Previous studies have shown that both ECM remodeling and cellular responses, such as astrogliosis, Müller cell activation, and microglial activation, contribute to biomechanical changes observed in myopic eyes [55,56]. These structural changes, including scleral thinning and collagen loss, lead to increased biomechanical susceptibility of the ONH [57]. (Since the biomechanical properties of the cornea reflect its ECM composition and may be related to those of the LC and peripapillary sclera, corneal biomechanics could serve as an indirect indicator of structural changes within the ONH.) Consistent with these findings, our results demonstrate that the stiffer group (defined by lower A1 deflection amplitude) was associated with younger age and reduced deep peripapillary vessel density (VD) (Table 1). This suggests that increased ocular tissue stiffness in myopic eyes may be linked to a greater vulnerability to glaucomatous damage at a younger age, potentially due to altered ECM profiles specific to myopic eyes. Furthermore, the biomechanical characteristics of stiffer tissue may affect deep vessel density, indicating a potential relationship between structural rigidity and vascular compromise in the ONH.

This study has a strength in assessing corneal biomechanics and vessel density together in myopic glaucoma patients, using Corvis ST and OCT-A, providing objective, quantitative results that enhance the reliability of the findings. Furthermore, the associations observed between corneal stiffness, vascular changes, and central visual field defects provide valuable insights for glaucoma risk assessment in myopic populations.

However, our study has several limitations. First, the relatively small sample size and the absence of longitudinal follow-up data limit the ability to evaluate the long-term impact of these factors on glaucoma progression. Additionally, while adjustments for age, IOP, and axial length were performed, potential confounders such as CCT and the use of topical antiglaucomatous medications may not have been fully accounted for. Moreover, given the relatively small sample size compared with the number of predictors, overfitting may have occurred in our multivariable models. Future prospective studies with larger cohorts and longitudinal data are needed to clarify the causal relationships and further explore the clinical implications of these findings.

## 5. Conclusions

In conclusion, corneal biomechanical properties were significantly related to peripapillary vessel density in glaucomatous myopic eyes, with different effects on superficial and deep vascular layers. Stiffer corneas were associated with lower deep VD and a greater risk of central VF defects, while more compliant corneas were associated with reduced superficial VD and increased posterior scleral deformation. These findings suggest that biomechanical properties may have potential clinical utility as a risk stratification tool for identifying myopic glaucoma patients at higher risk of central visual loss or structural progression. Nevertheless, given the limited sample size, these findings should be considered hypothesis-generating and will require validation in larger prospective studies.

## Figures and Tables

**Table 1:** Characteristics of study participants stratified based on A1 deflection amplitude.

Parameters	High A1 Deflection Amplitude (*n* = 21)	Low A1 Deflection Amplitude (*n* = 21)	*p*-Value
Age, years	57.2 ± 14.5	48.7 ± 9.8	**0.033**
Intraocular pressure, mmHg	16.1 ± 4.2	14.4 ± 2.4	0.102
Axial length, mm	26.5 ± 1.3	26.4 ± 1.1	0.813
Central corneal thickness	546.9 ± 32.0	536.8 ± 29.7	0.296
Disc–foveal angle, degree	7.1 ± 2.3	7.0 ± 4.8	0.943
Disc torsion, degree	18.0 ± 9.1	16.0 ± 10.1	0.510
PPA area	9953.1 ± 6686.6	9580.3 ± 6202.8	0.852
Peripapillary VD			
Superficial temporal VD	38.4 ± 10.5	40.6 ± 10.0	0.482
Superficial nasal VD	37.9 ± 11.0	42.8 ± 11.2	0.155
Deep VD	43.9 ± 12.9	36.2 ± 10.7	**0.042**
Superficial macular VD	31.1 ± 4.2	32.9 ± 3.5	0.137
Deep macular VD	34.9 ± 2.5	34.4 ± 2.1	0.440
Central 12-point MD sum in SITA 24-2, dB	−2.9 ± 4.2	−2.6 ± 3.4	0.762
MD in SITA 24-2, dB	−5.1 ± 5.0	−4.3 ± 4.9	0.633
PSD in SITA 24-2, dB	5.8 ± 4.0	4.7 ± 4.5	0.419
RNFL thickness	76.57 ± 11.9	77.43 ± 10.7	0.807

PPA = peripapillary atrophy, VD = vessel density, MD = mean deviation, SITA = Swedish interactive thresholding algorithm, PSD = pattern standard deviation, RNFL = retinal nerve fiber layer. Student’s *t*-test was used. Data are mean ± SD unless otherwise indicated. Factors with statistical significance are shown in bold.

**Table 2:** Correlations between biomechanical parameters of eyes and other factors.

		A1 Deformation Amp. [mm]	A1 Deflection Amp. [mm]	Whole-Eye Movement Max [mm]
Peripapillary VD				
Superficial temporal VD	r	−0.31	−0.09	−0.45
	*p*	**0.05**	0.58	**<0.01**
Deep VD	r	0.08	0.34	0.09
	*p*	0.63	**0.03**	0.57

VD = vessel density, A1 = applanation 1, Amp. = amplitude, Max = maximum. Spearman’s correlation coefficient was used. Factors with statistical significance are shown in bold.

**Table 3:** Factors associated with superficial peripapillary temporal vessel density.

	Univariate		Multivariate		Collinearity Statistics	
	B (95% CI)	*p*-Value	B (95% CI)	*p*-Value	Tolerance	VIF
Age	−0.351 (−0.575–−0.126)	**0.003**	−0.208 (−0.426–0.011)	0.062	0.89	1.12
CCT	0.038 (−0.066–0.143)	0.462				
IOP	−0.487 (−1.414–0.440)	0.295				
AXL	1.129 (−1.594–3.853)	0.407				
MD	0.707 (0.088–1.326)	**0.026**	0.665 (0.073–1.258)	**0.029**	0.81	1.24
RNFL thickness	0.397 (0.134–0.660)	**0.004**	0.125 (−0.161–0.410)	0.383	0.66	1.53
A1 DA [mm]	−221.830 (−442.544–−1.117)	**0.049**	−32.058 (−242.386–178.269)	0.759	0.80	1.25
Whole-eye movement max [mm]	−62.970 (−103.244–−22.696)	**0.003**	−42.340 (−76.404–−8.276)	**0.016**	0.95	1.05

CCT = central corneal thickness, IOP = intraocular pressure, AXL = axial length, MD = mean deviation, RNFL = retinal nerve fiber layer, A1 = applanation 1, DA = deformation amplitude, max = maximum, VIF = variance inflation factor. Factors with *p* < 0.1 in univariate analysis were included in multivariate analysis. Factors with statistical significance are shown in bold.

**Table 4:** Factors associated with deep peripapillary vessel density.

	Univariate		Multivariate		Collinearity Statistics	
	B (95% CI)	*p*-Value	B (95% CI)	*p*-Value	Tolerance	VIF
Age	−0.090 (−0.392–0.211)	0.549	−0.220 (−0.522–0.082)	0.148	0.90	1.12
CCT	0.010 (−0.117–0.138)	0.869				
IOP	0.230 (−0.904–1.363)	0.684	−0.312 (−1.463–0.838)	0.586	0.87	1.15
AXL	−0.509 (−3.827–2.808)	0.758	−1.647 (−4.887–1.593)	0.310	0.94	1.07
A1 DA [mm]	547.019 (62.377–1.0 × 10^3^)	**0.028**	745.458 (190.572–1.3 × 10^3^)	**0.01**	0.77	1.30

CCT = central corneal thickness, IOP = intraocular pressure, AXL = axial length, A1 = applanation 1, DA = deformation amplitude, VIF = variance inflation factor. Factors with *p* < 0.1 in univariate analysis were included in multivariate analysis. Factors with statistical significance are shown in bold.

**Table 5:** Factors associated with central scotoma presence.

	Univariate		Multivariate		Collinearity Statistics	
	Exp(B) (95% CI)	*p*-Value	Exp(B) (95% CI)	*p*-Value	Tolerance	VIF
Age	1.024 (0.974–1.077)	0.349				
CCT	0.983 (0.961–1.004)	0.110				
IOP	1.013 (0.841–1.220)	0.893	1.013 (0.803–1.277)	0.916	0.94	1.07
AXL	0.649 (0.369–1.139)	0.132	0.743 (0.381–1.449)	0.383	0.79	1.27
Peripapillary VD						
Superficial temporal VD	0.907 (0.838–0.982)	**0.017**	0.924 (0.849–1.006)	0.069	0.75	1.33
Whole-eye movement max [mm]	1.8 × 10^5^ (4.1 × 10^0^–7.9 × 10^9^)	**0.026**	1.1 × 10^3^ (0.003–3.9 × 10^8^)	0.280	0.62	1.60

CCT = central corneal thickness, IOP = intraocular pressure, AXL = axial length, VD = vessel density, max = maximum, VIF = variance inflation factor. Factors with *p* < 0.1 in univariate analysis were included in multivariate analysis. Factors with statistical significance are shown in bold.

**Table 6:** Factors associated with center 12-point MD from SITA 24-2.

	Univariate		Model 1			Model 2		
	B (95% CI)	*p*-Value	B (95% CI)	*p*-Value	VIF	B (95% CI)	*p*-Value	VIF
Age	0.051 (−0.039–0.141)	0.262						
CCT	0.003 (−0.035–0.042)	0.863						
IOP	−0.357 (−0.682–−0.032)	**0.032**	0.053 (−0.424–0.531)	0.822	2.96	−0.017 (−0.487–0.453)	0.942	2.78
AXL	0.107 (−0.899–1.113)	0.831	−0.379 (−1.368–0.610)	0.442	1.49	0.014 (−0.857–0.885)	0.974	1.12
Disc–foveal angle, degree	−0.346 (−0.647–−0.045)	**0.025**	−0.401 (−0.665–−0.138)	**0.004**	1.04	−0.411 (−0.679–−0.143)	**0.004**	1.04
Peripapillary VD								
Superficial temporal VD	0.115 (0.004–0.227)	**0.042**	0.101 (0.003–0.198)	**0.043**	1.06	0.123 (0.019–0.226)	**0.022**	1.17
HC deformation amp. [mm]	10.443 (1.577–19.308)	**0.022**				12.442 (−0.303–25.187)	**0.055**	2.70
HC deflection amp. [mm]	12.181 (3.703–20.660)	**0.006**	15.055 (1.694–28.415)	**0.028**	3.16			

CCT = central corneal thickness, IOP = intraocular pressure, AXL = axial length, VD = vessel density, HC = highest concavity, amp. = amplitude, VIF = variance inflation factor. Factors with *p* < 0.1 in univariate analysis were included in multivariate analysis. Factors with statistical significance are shown in bold.

## Data Availability

The data presented in this study are available on request from the corresponding author. The data are not publicly available due to patient privacy and ethical restrictions.

## Supplementary Materials

The following supporting information can be downloaded at https://www.mdpi.com/article/10.3390/jcm14186515/s1, Table S1: Corvis ST parameters, Table S2: Correlations between biomechanical parameters of eyes and other factors (expanded), Table S3: Factors associated with superficial peripapillary temporal vessel density (expanded), Table S4: Factors associated with deep peripapillary vessel density (expanded), Table S5: Factors associated with central scotoma presence (expanded), Table S6: Factors associated with center 12-point MD from SITA 24-2 (expanded).
