# Supplementary material for: Differential Effects of Corneal Biomechanics on Superficial and Deep Vessel Density and Their Association with Central Visual Function in Glaucoma Patients with Myopia
Source: J Clin Med. 2025 Sep 16;14(18):6515. doi: 10.3390/jcm14186515 (PMC12470511; doi:10.3390/jcm14186515)
Supplement: Supplementary file 1 [file jcm-14-06515-s001.zip › jcm-3868681-supplementary.pdf]

Supplementary Tables

Table S1. Corvis ST parameters

Parameters	Description
Deformation amplitude maximum (mm)	Maximum total deformation amplitude of the corneal apex (including whole-eye movement).
A1 deformation amplitude (mm)	Sum of the deflection amplitude and the WEM at A1
HC deformation amplitude (mm)	Corneal displacement at highest concavity
A2 deformation amplitude (mm)	Sum of the deflection amplitude and the WEM at A2
A1 deflection amplitude (mm)	Moving distance of the corneal apex from the initial position to that at the A1 time without WEM
HC deflection amplitude (mm)	Distance of the corneal apex movement from the initiation of the deformation to the highest concavity without WEM
A2 deflection amplitude (mm)	Moving distance of the corneal apex from the initial position to that at the A2 time without WEM
Deflection amplitude maximum (mm)	Maximum displacement of the corneal apex corrected for whole-eye movement during the deformation process
Whole-eye movement (mm)	The total amount of eye movement during the examination
DA ratio maximum (2 mm)	Ratio between the deformation amplitude at the apex and at 2 mm
Integrated radius (mm^-1)	Represents the amount of the corneal concave state over the time between A1 and A2
Stiffness parameter A1 (SP-A1) (mmHg/mm)	The resulting pressure on the cornea divided by the deflection amplitude at A1
Corvis Biomechanical Index (CBI)	Composite score indicating risk of keratoconus/ectasia.
Stress-Strain Index (SSI)	Dimensionless parameter reflecting overall material stiffness of the cornea.

A1 = applanation 1; A2 = applanation 2; DA = deformation amplitude; HC = highest concavity.

Table S2. Correlation between biomechanical parameters of eyes and other factors (expanded)

		Def. Amp. Max [mm]	A1 Deformation Amp. [mm]	HC Deformation Amp. [mm]	A2 Deformation Amp. [mm]	A1 Deflection Amp. [mm]	HC Deflection Amp. [mm]	A2 Deflection Amp. [mm]	Deflection Amp. Max [mm]	Whole Eye Movement Max [mm]	DA Ratio Max (1mm)	ARTh	Integrated Radius [mm^-1]	SP A1	SSI
Age	r	0.13	0.26	0.13	-0.14	0.26	0.08	-0.24	0.04	0.11	-0.07	0.17	-0.02	0.09	0.41
	p	0.43	0.09	0.43	0.37	0.10	0.60	0.13	0.79	0.51	0.66	0.28	0.90	0.58	**0.01**
CCT	r	-0.15	0.10	-0.15	0.17	0.36	-0.13	0.24	-0.11	-0.11	-0.04	0.31	-0.28	0.47	0.02
	p	0.36	0.53	0.36	0.29	**0.02**	0.43	0.13	0.51	0.50	0.80	**0.04**	0.07	**<0.01**	0.92
IOP	r	-0.75	0.64	-0.75	0.01	0.35	-0.74	0.08	-0.76	-0.10	-0.12	0.20	-0.51	0.79	0.17
	p	**<0.01**	**<0.01**	**<0.01**	0.97	**0.02**	**<0.01**	0.61	**<0.01**	0.53	0.47	0.20	**<0.01**	**<0.01**	0.29
Disc-foveal angle, degree	r	0.15	-0.08	0.15	0.31	-0.02	0.15	0.24	0.17	0.17	0.17	-0.01	0.07	-0.11	-0.11
	p	0.35	0.60	0.35	0.05	0.90	0.36	0.12	0.29	0.29	0.29	0.94	0.64	0.50	0.51
Disc torsion, degree	r	-0.08	-0.11	-0.08	0.12	0.12	-0.01	0.24	0.05	-0.13	0.05	0.29	0.12	0.13	-0.18
	p	0.62	0.50	0.62	0.45	0.46	0.96	0.13	0.77	0.41	0.77	0.07	0.47	0.43	0.27
PPA area	r	0.25	0.02	0.25	-0.10	0.12	0.31	-0.01	0.28	-0.18	-0.02	-0.01	0.11	-0.11	-0.27
	p	0.11	0.89	0.11	0.54	0.45	0.04	0.97	0.08	0.26	0.91	0.94	0.51	0.48	0.09
Peripapillary VD															
Superficial temporal VD	r	-0.06	-0.31	-0.06	-0.06	-0.09	0.12	0.16	0.09	-0.45	0.17	-0.11	-0.12	-0.10	-0.12
	p	0.73	0.05	0.73	0.72	0.58	0.44	0.30	0.56	<0.01	0.28	0.49	0.46	0.52	0.44
Superficial nasal VD	r	-0.02	-0.25	-0.02	0.01	-0.17	0.03	0.10	0.04	-0.16	-0.03	-0.21	-0.04	-0.11	-0.15
	p	0.90	0.11	0.90	0.95	0.29	0.85	0.52	0.81	0.31	0.87	0.19	0.80	0.48	0.33
Deep VD	r	0.05	0.08	0.05	0.19	0.34	0.04	0.13	-0.02	0.09	-0.12	0.05	-0.17	-0.13	0.01
	p	0.77	0.63	0.77	0.24	0.03	0.81	0.41	0.90	0.57	0.44	0.74	0.27	0.40	0.95
Superficial macular VD	r	0.00	-0.16	0.00	-0.13	-0.15	0.14	0.00	0.11	-0.30	0.05	-0.27	-0.13	-0.15	-0.07
	p	1.00	0.31	1.00	0.43	0.34	0.37	0.98	0.50	0.05	0.77	0.08	0.42	0.34	0.64
Deep macular VD	r	-0.13	0.00	-0.13	0.09	0.07	-0.11	0.02	-0.16	0.06	-0.18	-0.24	-0.40	0.02	0.22

	p	0.42	0.99	0.42	0.58	0.68	0.51	0.92	0.32	0.72	0.26	0.13	0.01	0.89	0.16
Central 12-point MD sum in SITA 24-2, dB	r	0.35	-0.15	0.35	-0.15	0.03	0.42	-0.05	0.41	-0.23	0.04	0.07	0.16	-0.21	-0.15
	p	0.02	0.35	0.02	0.34	0.85	0.01	0.74	0.01	0.14	0.80	0.68	0.31	0.18	0.33
MD in SITA 24-2, dB	r	0.18	-0.06	0.18	-0.09	-0.07	0.22	-0.07	0.18	-0.14	-0.08	-0.14	-0.14	-0.03	-0.11
	p	0.25	0.73	0.25	0.56	0.64	0.15	0.65	0.26	0.37	0.62	0.37	0.38	0.87	0.51
PSD in SITA 24-2, dB	r	-0.01	0.05	-0.01	0.03	0.19	-0.02	0.08	0.06	0.04	0.13	0.27	0.30	-0.10	0.06
	p	0.97	0.76	0.97	0.83	0.22	0.92	0.62	0.70	0.81	0.42	0.08	0.06	0.52	0.72

IOP = intraocular pressure, PPA = peripapillary atrophy, VD = vessel density, MD = mean deviation, SITA= Swedish interactive thresholding algorithm, PSD = pattern standard deviation, A1 = applanation 1, HC = highest concavity, A2 = applanation 2, DA = deformation amplitude, ARTh = Ambrósio relational thickness, SP = stiffness parameter, SSI = stress-strain index.

Spearman's correlation coefficient was used.

Factors with statistical significance are shown in bold.

Table S3. Factors associated with superficial peripapillary temporal vessel density (expanded)

	univariate		multivariate	
	B (95% CI)	*P*-Value	B (95% CI)	*P*-Value
Age	-0.351 (-0.575–-0.126)	**0.003**	-0.208 (-0.426–0.011)	0.062
CCT	0.038 (-0.066–0.143)	0.462		
IOP	-0.487 (-1.414–0.440)	0.295		
AXL	1.129 (-1.594–3.853)	0.407		
Disc-foveal angle, degree	-0.004 (-0.880–0.872)	0.993		
Disc torsion, degree	0.026 (-0.316–0.367)	0.881		
PPA area	0.000 (-0.001–0.000)	0.811		
MD	0.707 (0.088–1.326)	**0.026**	0.665 (0.073–1.258)	0.029
PSD	-0.761 (-1.495–-0.027)	**0.042**		
RNFL thickness	0.397 (0.134–0.660)	**0.004**	0.125 (-0.161–0.410)	0.383
Def. Amp. Max [mm]	-4.520 (-30.331–21.292)	0.725		
A1 Deformation Amp. [mm]	-221.830 (-442.544–-1.117)	**0.049**	-32.058 (-242.386–178.269)	0.759
HC Deformation Amp. [mm]	-4.520 (-30.331–21.292)	0.725		
A2 Deformation Amp. [mm]	-4.428 (-29.038–20.183)	0.718		
A1 Deflection Amp. [mm]	-117.293 (-541.814–307.228)	0.580		
HC Deflection Amp. [mm]	9.858 (-15.405–35.122)	0.435		
A2 Deflection Amp. [mm]	11.810 (-11.035–34.656)	0.302		
Whole Eye Movement Max [mm]	-62.970 (-103.244–-22.696)	**0.003**	-42.340 (-76.404–-8.276)	0.016
DA Ratio Max (2mm)	1.489 (-1.238–4.216)	0.276		
ARTh	-0.007 (-0.028–0.014)	0.490		
bIOP	-0.439 (-1.468–0.589)	0.393		
Integrated Radius [mm^-1]	-0.848 (-3.135–1.439)	0.458		
SP A1	-0.057 (-0.233–0.119)	0.518		
CBI	3.699 (-9.157–16.555)	0.564		
SSI	-5.478 (-19.628–8.672)	0.439		

IOP = intraocular pressure, AXL = axial length, PPA = peripapillary atrophy, MD = mean deviation, PSD = pattern standard deviation, A1 = applanation 1, HC = highest concavity, A2 = applanation 2, DA = deformation amplitude, ARTh = Ambrósio relational thickness, SP = stiffness parameter, CBI = corvis biomechanical index, SSI = stress-strain index

Factors with P < .1 in univariate analysis were included in multivariate analysis.

Factors with statistical significance are shown in bold.

Table S4. Factors associated with deep peripapillary vessel density (expanded)

	univariate		multivariate	
	B (95% CI)	P-Value	B (95% CI)	P-Value
Age	-0.090 (-0.392–0.211)	0.549	-0.220 (-0.522–0.082)	0.148
CCT	0.010 (-0.117–0.138)	0.869		
IOP	0.230 (-0.904–1.363)	0.684	-0.312 (-1.463–0.838)	0.586
AXL	-0.509 (-3.827–2.808)	0.758	-1.647 (-4.887–1.593)	0.310
Disc-foveal angle, degree	0.158 (-0.900–1.215)	0.765		
Disc torsion, degree	-0.073 (-0.485–0.340)	0.724		
PPA area	0.000 (-0.001–0.001)	0.721		
MD	-0.436 (-1.246–0.319)	0.239		
PSD	0.381 (-0.546–1.307)	0.411		
RNFL thickness	-0.075 (-0.428–0.277)	0.667		
Def. Amp. Max [mm]	4.446 (-26.774–35.666)	0.775		
A1 Deformation Amp. [mm]	68.064 (-211.337–347.466)	0.625		
HC Deformation Amp. [mm]	4.446 (-26.774–35.666)	0.775		
A2 Deformation Amp. [mm]	17.384 (-11.894–46.663)	0.237		
A1 Deflection Amp. [mm]	547.019 (62.377–1.0E+03)	**0.028**	745.458 (190.572–1.3E+03)	**0.01**
HC Deflection Amp. [mm]	3.633 (-27.122–34.389)	0.813		
A2 Deflection Amp. [mm]	11.423 (-16.330–39.176)	0.41		
Whole Eye Movement Max [mm]	15.489 (-38.712–69.690)	0.567		
DA Ratio Max (2mm)	-1.276 (-4.597–2.046)	0.442		
ARTh	0.004 (-0.021–0.030)	0.741		
bIOP	0.297 (-0.953–1.548)	0.633		
Integrated Radius [mm^-1]	-1.502 (-4.244–1.240)	0.275		
SP A1	-0.089 (-0.301–0.123)	0.399		
CBI	-3.066 (-18.642–12.511)	0.693		
SSI	0.545 (-16.691–17.781)	0.949		

IOP = intraocular pressure, AXL = axial length, PPA = peripapillary atrophy, MD = mean deviation, PSD = pattern standard deviation, A1 = applanation 1, HC = highest concavity, A2 = applanation 2, DA = deformation amplitude, ARTh = Ambrósio relational thickness, SP = stiffness parameter, CBI = corvis biomechanical index, SSI = stress-strain index

Factors with $P < .1$ in univariate analysis were included in multivariate analysis.

Factors with statistical significance are shown in bold.

Table S5. Factors associated with central scotoma presence (expanded)

	univariate		multivariate	
	Exp(B) (95% CI)	*P*-Value	Exp(B) (95% CI)	*P*-Value
Age	1.024 (0.974–1.077)	0.349		
CCT	0.983 (0.961–1.004)	0.110		
IOP	1.013 (0.841–1.220)	0.893	1.013 (0.803–1.277)	0.916
AXL	0.649 (0.369–1.139)	0.132	0.743 (0.381–1.449)	0.383
Disc-foveal angle, degree	1.089 (0.897–1.321)	0.388		
Disc torsion, degree	1.003 (0.938–1.073)	0.932		
PPA area	1.000 (1.000–1.000)	0.929		
Peripapillary VD				
Superficial temporal VD	0.907 (0.838–0.982)	**0.017**	0.924 (0.849–1.006)	0.069
Superficial nasal VD	1.020 (0.964–1.081)	0.491		
Deep VD	1.009 (0.958–1.063)	0.739		
Superficial macular VD	0.869 (0.727–1.038)	0.121		
Deep macular VD	0.987 (0.747–1.303)	0.924		
Def. Amp. Max [mm]	2.128 (0.013–343.712)	0.771		
A1 Deformation Amp. [mm]	5.3E+09 (4.2E-12–6.8E+30)	0.366		
HC Deformation Amp. [mm]	2.128 (0.013–343.712)	0.771		
A2 Deformation Amp. [mm]	32.431 (0.046–22,858.737)	0.298		
A1 Deflection Amp. [mm]	1.4E+18 (9.6E-22–1.9E+57)	0.364		
HC Deflection Amp. [mm]	0.265 (0.002–40.875)	0.606		
A2 Deflection Amp. [mm]	0.620 (0.008–50.909)	0.832		
Whole Eye Movement Max [mm]	1.8E+05 (4.1E+00–7.9E+09)	**0.026**	1.1E+03 (0.003–3.9E+08)	0.280
DA Ratio Max (2mm)	0.533 (0.158–1.800)	0.311		
ARTh	0.998 (0.994–1.002)	0.389		
Integrated Radius [mm^-1]	0.922 (0.589–1.445)	0.724		
SP A1	0.981 (0.946–1.017)	0.288		
CBI	2.816 (0.182–43.532)	0.459		
SSI	8.220 (0.329–205.084)	0.199		

IOP = intraocular pressure, AXL = axial length, PPA = peripapillary atrophy, MD = mean deviation, PSD = pattern standard deviation, A1 = applanation 1, HC = highest concavity, A2 = applanation 2, DA = deformation amplitude, ARTh = Ambrósio relational thickness, SP = stiffness parameter, CBI = corvis biomechanical index, SSI = stress-strain index

Factors with P < .1 in univariate analysis were included in multivariate analysis.

Factors with statistical significance are shown in bold.

Table S6. Factors associated with center 12 points MD from SITA 24-2 (expanded)

	univariate		model 1		model 2	
	B (95% CI)	*P*-Value	B (95% CI)	*P*-Value	B (95% CI)	*P*-Value
Age	0.051 (-0.039–0.141)	0.262				
CCT	0.003 (-0.035–0.042)	0.863				
IOP	-0.357 (-0.682–-0.032)	**0.032**	0.053 (-0.424–0.531)	0.822	-0.017 (-0.487–0.453)	0.942
AXL	0.107 (-0.899–1.113)	0.831	-0.379 (-1.368–0.610)	0.442	0.014 (-0.857–0.885)	0.974
**Disc-foveal angle, degree**	-0.346 (-0.647–-0.045)	**0.025**	-0.401 (-0.665–-0.138)	**0.004**	-0.411 (-0.679–-0.143)	**0.004**
Disc torsion, degree	-0.034 (-0.159–0.091)	0.583				
PPA area	0.000 (0.000–0.000)	0.909				
Peripapillary VD						
**Superficial temporal VD**	0.115 (0.004–0.227)	**0.042**	0.101 (0.003–0.198)	**0.043**	0.123 (0.019–0.226)	**0.022**
Superficial nasal VD	0.023 (-0.082–0.129)	0.658				
Deep VD	-0.042 (-0.138–0.054)	0.383				
Superficial macular VD	0.244 (-0.049–0.538)	0.101				
Deep macular VD	0.175 (-0.340–0.691)	0.495				
Def. Amp. Max [mm]	10.443 (1.577–19.308)	0.022				
A1 Deformation Amp. [mm]	-39.621 (-123.618–44.377)	0.346				
HC Deformation Amp. [mm]	10.443 (1.577–19.308)	**0.022**			12.442 (-0.303–25.187)	**0.055**
A2 Deformation Amp. [mm]	-4.256 (-13.186–4.674)	0.341				
A1 Deflection Amp. [mm]	14.956 (-141.136–171.049)	0.847				
HC Deflection Amp. [mm]	12.181 (3.703–20.660)	**0.006**	15.055 (1.694–28.415)	**0.028**		
A2 Deflection Amp. [mm]	-1.410 (-9.883–7.063)	0.738				
Whole Eye Movement Max [mm]	-11.957 (-28.006–4.092)	0.140				
DA Ratio Max (2mm)	0.131 (-0.882–1.145)	0.795				
ARTh	0.002 (-0.006–0.009)	0.679				
Integrated Radius [mm^-1]	0.423 (-0.410–1.256)	0.311				
SP A1	-0.042 (-0.106–0.021)	0.184				
CBI	0.089 (-4.642–4.819)	0.970				
SSI	-2.505 (-7.668–2.658)	0.333				

IOP = intraocular pressure, AXL = axial length, PPA = peripapillary atrophy, MD = mean deviation, PSD = pattern standard deviation, A1 = applanation 1, HC = highest concavity, A2 = applanation 2, DA = deformation amplitude, ARTh = Ambrósio relational thickness, SP = stiffness parameter, CBI = corvis biomechanical index, SSI = stress-strain index

Factors with P < .1 in univariate analysis were included in multivariate analysis.

Factors with statistical significance are shown in bold.
